# Precision spatiotemporal analysis of large-scale compound–protein interactions through molecular dynamics simulation

**DOI:** 10.1093/pnasnexus/pgaf094

**Published:** 2025-03-22

**Authors:** Shigeyuki Matsumoto, Yuta Isaka, Ryo Kanada, Biao Ma, Mitsugu Araki, Shuntaro Chiba, Atsushi Tokuhisa, Hiroaki Iwata, Shoichi Ishida, Yoshinobu Akinaga, Kei Terayama, Ryosuke Kojima, Yohei Harada, Kazuhiro Takemura, Teruki Honma, Akio Kitao, Yasushi Okuno

**Affiliations:** Graduate School of Medicine, Kyoto University, 53 Shogoin-Kawaharacho, Sakyo-ku, Kyoto 606-8507, Japan; RIKEN Center for Computational Science, 7-1-26 Minatojima-minamimachi, Chuo-ku, Kobe, Hyogo 650-0047, Japan; RIKEN Center for Computational Science, 7-1-26 Minatojima-minamimachi, Chuo-ku, Kobe, Hyogo 650-0047, Japan; RIKEN Center for Computational Science, 7-1-26 Minatojima-minamimachi, Chuo-ku, Kobe, Hyogo 650-0047, Japan; Graduate School of Medicine, Kyoto University, 53 Shogoin-Kawaharacho, Sakyo-ku, Kyoto 606-8507, Japan; HPC- and AI-Driven Drug Development Platform Division, RIKEN Center for Computational Science, 1-7-22 Suehiro-cho, Tsurumi-ku, Yokohama, Kanagawa 230-0045, Japan; RIKEN Center for Computational Science, 7-1-26 Minatojima-minamimachi, Chuo-ku, Kobe, Hyogo 650-0047, Japan; Graduate School of Medicine, Kyoto University, 53 Shogoin-Kawaharacho, Sakyo-ku, Kyoto 606-8507, Japan; Graduate School of Medical Life Science, Yokohama City University, 1-7-29, Suehiro-cho, Tsurumi-ku, Kanagawa 230-0045, Japan; RIKEN Center for Computational Science, 7-1-26 Minatojima-minamimachi, Chuo-ku, Kobe, Hyogo 650-0047, Japan; Graduate School of Medical Life Science, Yokohama City University, 1-7-29, Suehiro-cho, Tsurumi-ku, Kanagawa 230-0045, Japan; Graduate School of Medicine, Kyoto University, 53 Shogoin-Kawaharacho, Sakyo-ku, Kyoto 606-8507, Japan; Graduate School of Medicine, Kyoto University, 53 Shogoin-Kawaharacho, Sakyo-ku, Kyoto 606-8507, Japan; School of Life Sciences and Technology, Institute of Science Tokyo, 2Chome 12-1, Ookayama, Meguro-ku, Tokyo 152-8550, Japan; HPC- and AI-Driven Drug Development Platform Division, RIKEN Center for Computational Science, 1-7-22 Suehiro-cho, Tsurumi-ku, Yokohama, Kanagawa 230-0045, Japan; School of Life Sciences and Technology, Institute of Science Tokyo, 2Chome 12-1, Ookayama, Meguro-ku, Tokyo 152-8550, Japan; Graduate School of Medicine, Kyoto University, 53 Shogoin-Kawaharacho, Sakyo-ku, Kyoto 606-8507, Japan; RIKEN Center for Computational Science, 7-1-26 Minatojima-minamimachi, Chuo-ku, Kobe, Hyogo 650-0047, Japan

**Keywords:** large-scale molecular dynamics simulation, spatiotemporal analysis, drug screening, supercomputer, COVID-19

## Abstract

Biological systems are composed of and regulated by intricate and diverse biomolecular interactions. Experimental and computational approaches have been developed to elucidate the mechanisms of these interactions; however, owing to cost, time, and accuracy issues, large-scale spatiotemporal analyses of molecular pairs remain challenging. Thus, the molecular recognition mechanisms underlying these diverse interactions remain unclear. We successfully simulated the large-scale molecular dynamics (MD) of 4,275 protein–compound pairs by combining a method to accelerate the MD simulations with the supercomputer Fugaku. Our spatiotemporal analysis of generated big MD data revealed universal features underlying molecular recognition and binding processes. This study expands our understanding of the concept of MD simulations from a technique to investigate the dynamic properties of individual protein–drug pairs to an approach to perform large-scale spatiotemporal analysis and compound screening. This study opens an avenue in biological research for subsequent drug discovery.

**Significance Statement**

Understanding molecular interactions at the atomic level is crucial for biological research and drug discovery, yet large-scale analyses are hindered by high costs, long-time requirements, and issues with accuracy. We introduce a large-scale precision spatiotemporal analysis using accelerated molecular dynamics (MD) simulations on the supercomputer Fugaku, achieving an unprecedented study of 4,275 compound–protein pairs. Our research reveals universal molecular recognition features that govern stable interactions and binding processes over broad timescales, shifting MD simulation from a tool for analyzing individual interactions to a method for large-scale spatiotemporal analysis and compound screening. Our findings advance biological research and enhance the role of MD simulations in drug discovery by providing essential structural and dynamic insights.

## Introduction

Various biomolecular interactions govern biological phenomena. Elucidating the molecular mechanisms underlying these diverse intermolecular interactions is essential for understanding and regulating biological systems. Atomic-level molecular interactions have been experimentally investigated using various biophysical approaches, such as nuclear magnetic resonance, X-ray crystallography, and cryogenic electron microscopy. However, large-scale analyses of molecular interactions are impractical because these experimental approaches are typically expensive and time-consuming. High-throughput screening has been used to explore active compounds from a large chemical compound library through biochemical assays to evaluate interactions on a large scale. However, high-throughput screening does not provide precise structural and dynamical information, which is fundamental for understanding molecular interactions. Thus, the spatiotemporal molecular recognition mechanisms underlying the diverse sets of interactions remain to be elucidated.

Recently, substantial interest has increased in the application of computational approaches as low-cost and simple methods for estimating molecular interactions. The computational approaches can be divided into the following categories: docking simulations based on 3D structures of proteins and AI approaches that leverage machine learning models trained based on existing experimental data (1–3). In docking simulations, the 3D structures of drug–protein complexes and their binding affinities are estimated. In recent years, ultra-large-scale docking simulations for virtual compound screening on high-performance computing, identifying inhibitors with high affinities (4). Large-scale docking simulations are typically performed using fixed conformations of the target proteins to reduce computational costs, i.e. rigid-body docking simulations. However, because molecular interactions are fundamentally dynamic processes, the prediction accuracy of the simulation is significantly impaired by the lack of dynamic properties. Meanwhile, compared with docking simulations, affinity predictions using an AI approach are faster. However, the predictive accuracy of these methods relies on existing real-world experimental data used for training, which may be biased or incomplete; predicting interactions for target proteins with little or no existing binding data is nearly impossible. Furthermore, since both the virtual methods typically ignore the dynamic behaviors of proteins and compounds, it is difficult to capture precise complex structures and the nature of the molecular interactions in solution.

Molecular dynamics (MD) simulations can capture atomic-level dynamic processes without prior knowledge by generating time-evolved atomic trajectories of molecular systems via repeatedly calculating the forces between atoms in solution to update the atomic positions (5–7). The previous MD studies for protein–drug pairs have demonstrated that the affinity predictions considering the dynamic behaviors is highly accurate, and furthermore, the obtained MD data provide precise structural information, including binding pathways and interaction modes (8–12). However, the computational cost of MD simulations is several thousand times higher than that of conventional docking simulations and AI approaches. Consequently, large-scale MD simulations for various pairs have been impractical despite substantial interest.

**Fig. 1.**
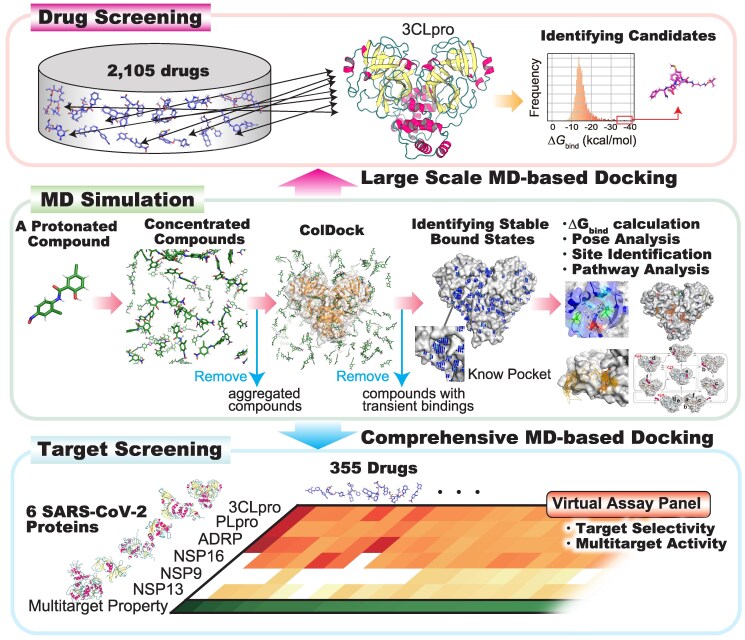
Overall workflow of spatiotemporal analysis using MD simulations. The stable bound states were identified in the MD simulation phase (Fig. S1), and their complex structures were used for subsequent analyses, such as Δ*G*_bind_ calculation, pose analysis, site identification, and pathway analysis. During this analysis, the aggregated compounds under high-concentration conditions (200 mM) and the compounds without stable bindings were excluded. Following this process, MD-based spatiotemporal analysis was performed. In evaluating molecular interactions of drugs toward six SARS-CoV-2 proteins, the target selectivity and multitarget activity for each drug were determined based on the Δ*G*_bind_ values. In the screening, drugs estimated to exhibit high binding affinities toward 3CLpro and their binding poses were identified.

In this study, we conducted the first successful large-scale spatiotemporal analysis of 4,275 protein–compound pairs by combining a method to accelerate MD simulation with Fugaku (13), one of the world's leading supercomputers (Fig. 1). We selected SARS-CoV-2 proteins as targets for our approach. SARS-CoV-2 proteins served as the study object to demonstrate the power of the proposed MD-based approach given the recent extensive research on COVID-19. By comprehensively analyzing the generated big MD data, we uncovered the universal features that underlie the diverse molecular protein interactions, including crucial interaction modes that govern the stable binding of drugs and proteins, directional preferences in local binding pathways, drug-acceptable pockets on the protein surface overall, and their roles during the binding process on a long time scale. In this paper, we demonstrate the potential of this large-scale MD-based approach for drug screening. These achievements pave the way toward new approaches to molecular biology research and subsequent drug discovery.

## Results

### Workflow of spatiotemporal analysis using MD simulations

The binding process between compounds and proteins typically is an event occurring on a timescale of microseconds or longer. Thus, it is difficult to capture it using general-purpose computers or conventional MD techniques. To address this challenge, a special-purpose supercomputer for MD simulations and various MD-accelerating algorithms have been developed. The main goal of our study is to perform a large-scale spatiotemporal analysis through MD-based docking simulations for thousands of compound–protein combinations, which was not achievable with conventional MD simulations due to the computational costs. In this effort, we utilized supercomputer Fugaku comprising ∼160,000 nodes and adopted an ensemble MD approach. We performed ten relatively short MD calculations in parallel for each compound–protein pair (totaling 1 μs: 100 ns × 10 parallel computations). Furthermore, to compensate for the limited sampling within the short MD simulations, we employed the concentrated ligand docking (ColDock) method (14), a technique designed to accelerate intermolecular collision occurring on the overall protein surface by simulating under conditions of high ligand concentration (Movies S1 and S2). In this study, calculations for a total of 4,275 protein–compound pairs (equivalent to 42,750 runs) were performed using ultra-parallel computing on the abundant nodes of Fugaku.

**Fig. 2.**
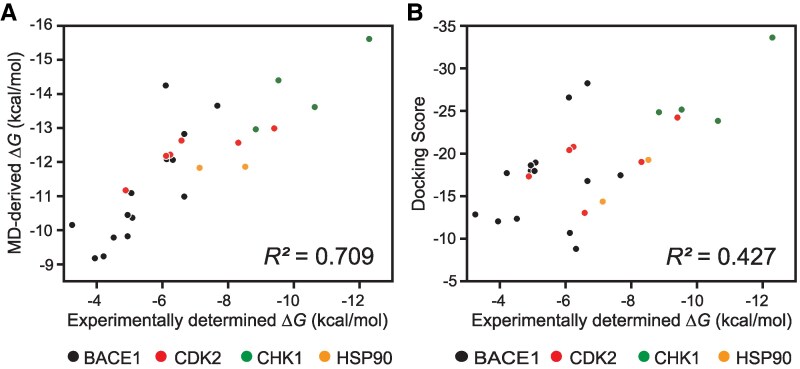
Benchmark experiments for affinity prediction with MD-based docking simulations. A and B) Correlation between the experimentally determined binding affinity and the Δ*G*_bind_ value derived from the MD simulations (A) and the docking score derived from a conventional docking simulation (B). Each point represents a compound–protein pair, and the points are colored according to the protein. The coefficient of determination (*R*^2^) across proteins is indicated in each plot.

To validate the performance of ColDock in assessing the compound–protein binding affinity, we employed four proteins (BACE1, CHK1, HSP90, and CDK2) as the benchmarks, since they had experimentally determined binding activity data available for multiple compounds (a total of 40, with *K*_d_/*K*_i_ values ranging from 10^−3^ to 10^−10^ M). Figure 1 illustrates the workflow from preparation of the system for ColDock to calculation of the binding free energy (Δ*G*_bind_). In this workflow, after removing the compounds that aggregate under the high-concentration conditions in the absence of proteins from the prepared datasets, Δ*G*_bind_ is estimated from the stable bound states observed on the molecular surface during the MD simulation. To calculate Δ*G*_bind_, it is essential to define the bound state of compounds in the calculated MD trajectories. Here, we defined the bound state as remaining in the target pocket for 30 ns or longer (Fig. S1). For the calculation of Δ*G*_bind_, we employed the linear interaction energy (LIE) approach (15), which calculates it by explicitly considering ligand-bound and nonbound states in solution. In practice, applying the workflow to the benchmark set of 40 compound–protein pairs, five compounds showed aggregation due to high-concentration conditions, and nine compounds were unable to observe the stable bound state within a total calculation time of 1 μs. Finally, Δ*G*_bind_ were calculated for the 26 drugs. Appropriate scaling factors *α* and *β* for $\Delta V^{\mathrm{vdw}}$ and $\Delta V^{\mathrm{ele}}$, respectively, in the Δ*G*_bind_ calculation need to be determined via fitting to experimental data. We aimed to estimate the Δ*G*_bind_ values for target proteins with few known inhibitors and the limited experimentally determined complex structures. Therefore, obtaining the scaling factors that can be commonly applied across multiple proteins is preferable. To achieve this, we explored the *α* and *β* values that relatively most accurately reproduced experimental values across four benchmark proteins (26 protein–compound pairs) by performing a grid search for the values ranging from 0.05 to 1.00 in increments of 0.05 (Fig. S2). As a result, the values of α=1.00 and β=0.05 were identified as offering the strongest correlation with the experimental values. The Δ*G*_bind_ values calculated with the determined scaling factors exhibited a strong correlation with the experimental values (Fig. 2A, coefficient of determination *R*^2^ = 0.709). The Δ*G*_bind_ derived from LIE approach tends to provide overall higher estimates due to the omission of the conformational entropy loss, but the strong correlation indicates the effectiveness of this approach in MD-based screening to identify compounds with relatively higher affinities. Furthermore, our MD approach is noteworthy for its generalizability; the correlation was maintained across the four proteins and various compounds, demonstrating that the method's accuracy does not depend on the protein or compound (Fig. 2A). In contrast, the *R*^2^ value between the docking score derived from rDock (16), a conventional rigid-body docking software, and experimental values was 0.427 (Fig. 2B). This result highlights the importance of considering the flexibility of proteins in solution for accurately assessing affinity. These findings demonstrate the effectiveness of the present approach, in which ColDock accelerates binding events that typically require extensive computational cost to observe them in the conventional MD simulations by ensuring quantitative capability for assessing the compound–protein binding affinity. Furthermore, this method allows for scalability to evaluate a large number of compounds simply by increasing the number of parallel computations on a supercomputer. These results prompted us to subsequently perform spatiotemporal analysis of the large-scale compound–protein combinations to explore its practical use.

### Evaluation multiple interactions between 355 drugs and six SARS-CoV-2 proteins using MD simulation

**Fig. 3.**
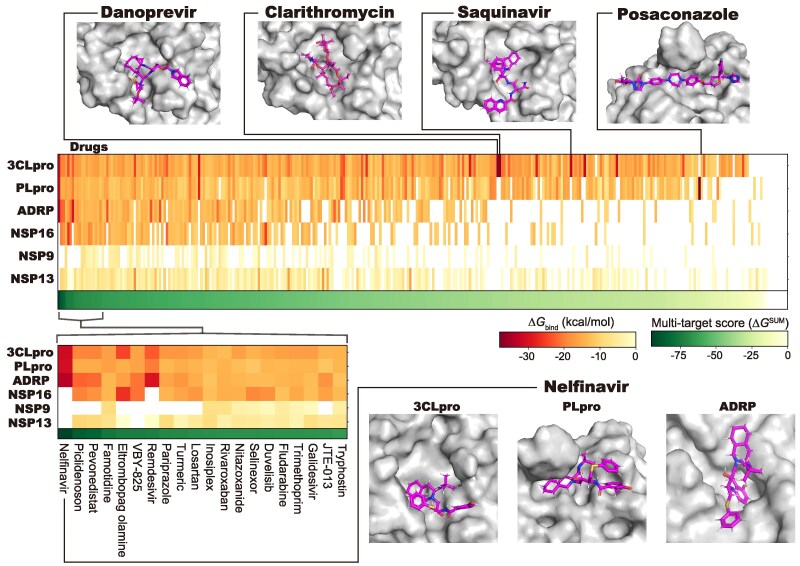
MD-based evaluation of target selectivity and multitarget activities of drugs investigated in previous preclinical or clinical studies. The Δ*G*_bind_ values of the different combinations and the multitarget score for each compound are shown in heat maps with color bars. In the heat map, the horizontal axis represents the screened drugs, and the vertical axis represents the proteins. Combinations for which no binding was observed are shown in white. The bottom left panel shows the heat map of the 20 drugs with the highest multitarget scores. The representative binding poses of the drugs exhibiting high selectivity or multitarget capability are shown. In the 3D structures, the proteins and the bound drugs are represented by surface and stick models, respectively.

Numerous preclinical and clinical studies have been carried out in response to COVID-19 pandemic. However, the target molecules of the evaluated drugs have remained unclear in many cases, posing substantial challenges in improving therapeutic efficacy, addressing mutations, and preventing unexpected side effects. Thus, using the current approach, we evaluated the target molecules of 355 drugs, which were derived from a public data repository (https://ghddi-ailab.github.io/Targeting2019-nCoV) and were previously investigated in preclinical or clinical studies, for six SARS-CoV-2 proteins involved in viral replication (3CLpro, PLpro, ADRP, NSP16, NSP9, and NSP13; 2,130 compound–protein pairs in total). Among the 355 evaluated drugs, 309 drugs possessed stable binding poses, and the Δ*G*_bind_ values were calculated for all combinations with the six target proteins, i.e. virtual panel assay (Table S1). The comprehensive spectrum of calculated binding activities illustrated distinct profiles for each compound and protein together with the complex structures (Fig. 3).

In drug development, target selectivity is crucial for preventing unexpected side effects. Therefore, we evaluated the target selectivity of each drug by examining the top-ranked drugs for each protein. Danoprevir, clarithromycin, and saquinavir exhibited high selectivity for 3CLpro with lower than −30 kcal/mol of the Δ*G*_bind_ values, whereas they did not bind to the other proteins (Fig. S3). Similarly, posaconazole, which attained a Δ*G*_bind_ value of −37.898 kcal/mol, was identified as a drug with high selectivity for PLpro. Nelfinavir and lopinavir were predicted to bind with high affinity to 3CLpro (−31.2 kcal/mol) and PLpro (−30.7 kcal/mol); thus, viral replication should be effectively inhibited by their synergistic effect against protease activity. Nelfinavir and eltrombopag olamine showed the most favorable Δ*G*_bind_ values for ADRP (−33.5 kcal/mol) and NSP16 (−26.6 kcal/mol), respectively. However, the selectivity of these drugs was low because they also bound to 3CLpro and PLpro. No drugs with notably high Δ*G*_bind_ values were identified for NSP9 or NSP13.

**Fig. 4.**
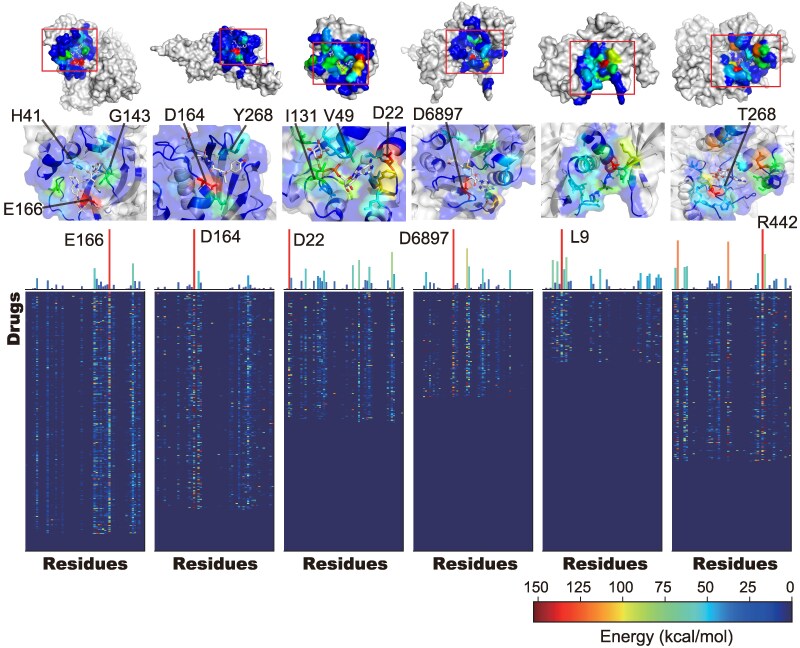
Comprehensive PLIF analysis based on the evaluated drugs. For each of the six SARS-CoV-2 proteins, the PLIF values for each drug were calculated using the complex structures obtained by the MD simulations and are visualized as heat maps. The horizontal axis represents the residues in each protein, and the vertical axis represents the 309 drugs. In the upper part of the heat maps, the sum of the PLIF values is shown as a bar graph and mapped on the 3D structure. In the 3D structures, the known inhibitors and the residues exhibiting higher energies are represented by stick models, and the residues involved in prominent interactions with the known ligands are labeled.

While selectivity is important for preventing unwanted side effects, broad-spectrum or multitarget activity is often desirable for antiviral drugs because the mutation rates of target proteins are high in this context. Through broad-spectrum activity, the drugs remain effective even if one or more target sites acquire a mutation. Hence, we assessed the multitarget capability of each drug by calculating the total Δ*G*_bind_ values for the six proteins as the multitarget score (Fig. 4). We ranked the drugs based on their multitarget scores and found distinct drugs from the top-ranking drugs for each protein (Figs. 3 and S3, the MD videos of the top-ranking drugs based on the multitarget scores can be found at https://clinfo.med.kyoto-u.ac.jp/md_based_screening/si/si1.html). By comparing saquinavir and nelfinavir, which are antiviral drugs with multitarget scores of −32.2 and −95.4 kcal/mol, respectively, we found that nelfinavir exhibited a broader spectrum and may be preferable as an antiviral agent. Eltrombopag olamine, which has a high multitarget score (−74.0 kcal/mol), has previously been reported to exhibit antiviral activity in various anti-SARS-CoV-2 assays (17), which is consistent with the high score observed. Additionally, drugs that were previously reported to exhibit antiviral activity, such as VBY-825 (18, 19) and remdesivir (20), were included in the top-ranked drugs. These drugs could be clinically favorable for treating COVID-19 since the multitarget property is often desirable for antiviral drug development.

Compared with compounds with high selectivity, compounds with multitarget properties are more difficult to rationally develop because it involves scenarios beyond simply improving the binding activity toward a specific target. Intriguingly, compared with drugs with high selectivity for a specific target, drugs with high multitarget scores tended to exhibit lower Δ*G*_bind_ values (Fig. S4). Based on this observation, a trade-off occurs between the multitargeting capability and target selectivity of the drugs. This finding could provide a clue to develop new drugs with multitarget capabilities.

### Unveiling crucial interaction modes that govern the stable drug–protein interactions using big MD data

Obtaining structural information related to interaction modes of drugs at atomic levels is crucial for efficiently developing drugs, and the present MD data precisely provide this information for the drugs with chemically diverse structures. Then, we comprehensively analyzed the interaction modes of the drugs for the six SARS-CoV-2 proteins. Protein‒ligand interaction fingerprints (PLIFs), which encode interaction pairs and the types formed between protein residues and the bound ligand, were calculated for all complex structures of the 309 drugs that exhibited stable bound states (Fig. S5). The averaged PLIFs illustrated distinct profiles for each protein (heat maps in Fig. 4). The PLIFs of each drug could be diverse since their chemical structures are irrelevant, but interestingly, the residues that contributed to drug recognition (key residues) were commonly shared across different drugs, as demonstrated by the total PLIFs for all the drugs (bar graphs in Figs. 4 and S6). The key residues identified corresponded well with those found in crystal structures complexed with the known ligand (tertiary structures in Fig. 4). The Glu166 residue, which is frequently involved in the formation of hydrogen bonds with drugs in PLIF analyses for 3CLpro, has been reported to play a central role in ligand recognition (21) (PDB entry; 6Y2G). The Asp164 prominent residues in the PLIF analysis for PLpro acted as hydrogen bond acceptors to recognize known inhibitory peptides (22) (PDB entry; 6WX4). Similarly, Asp22 in ADRP and Asp6897 and Asp6912 in NSP16 participated in the recognition of known ligands (23, 24) (PDB entry; 6W02 and 6W4H, respectively). In addition to the residues found in the previous experiments, several residues not involved in the recognition of known ligands were identified as key residues, such as Arg189 in 3CLpro and Arg166 in PLpro. Based on these analyses, utilizing big MD data offers a unique approach to comprehensively identify potential residues at target pockets; in contrast, these analyses cannot be achieved by conventional approaches.

### Illustrating the binding pathways of drugs on the overall protein surface with large-scale MD data analysis

In addition to determining the structures of stable drug‒protein complexes, analyzing the binding pathways is essential for clarifying molecular interactions because the binding event is a dynamic process in which drugs contact the protein, reach the target pocket, and form stable interactions. In ColDock, the drug‒protein bindings occur on the overall protein surface; thus, we adopted the following approaches to investigate the underlying pathways for the drugs used in preclinical and clinical studies: one approach focused on local binding pathways around the target pocket and another focused on global binding pathways over the entire protein.

**Fig. 5.**
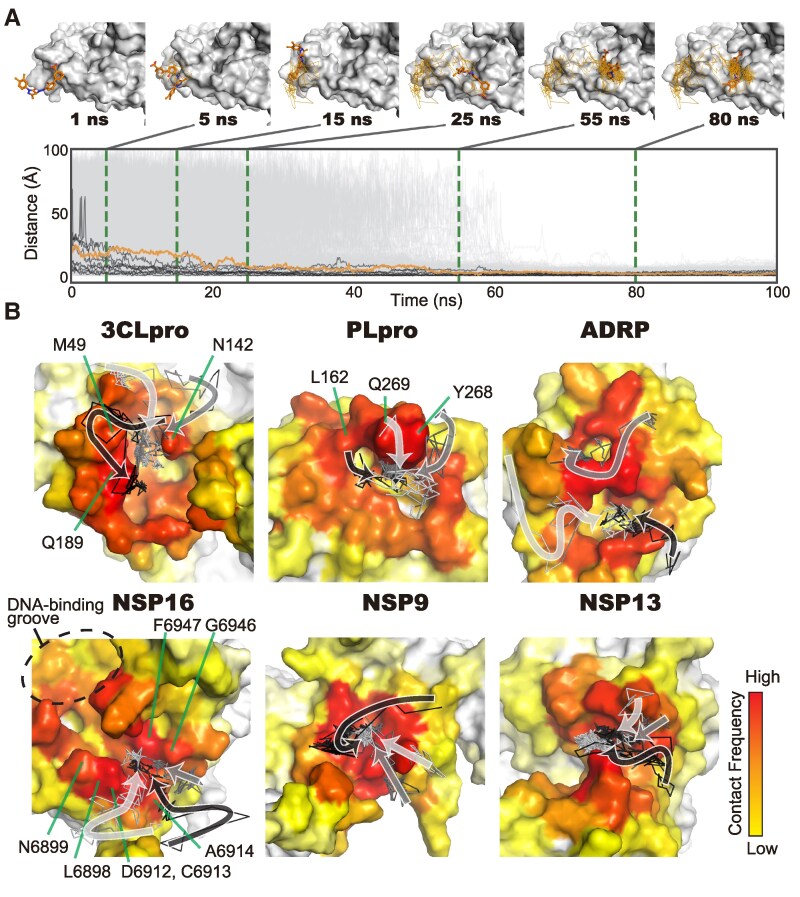
Comprehensive analysis of local binding pathways near the target pockets. A) Approaching the target pocket of the evaluated drugs during MD simulations. The distance to the target pocket in 3CLpro, a major therapeutic target, over time is shown. The vertical axis represents distance from the target pocket, and the horizontal axis represents time in units of ns. The distance transitions illustrating gradual approaches to the target pocket for all drugs are shown by the gray lines, and those for the representative drug, eltrombopag olamine, are shown by the black and orange lines. The upper panel shows the representative complex structures found in the pathway indicated by the orange line. The structures are extracted at the times indicated by the green dashed lines. In the 3D structures, the bound ligands are represented by stick models, and the binding processes from 1 ns are indicated by the orange lines. B) Visualization of the residues involved in the contacts with drugs during the binding processes. Representative binding processes of the top three clusters derived from the clustering of the binding pathways are indicated by the solid black, dark gray, and light gray lines. Their approximate trajectories are indicated by arrows. The residues involved in the directional preferences are indicated by green lines and labeled.

First, we investigated the local binding pathways near the target pockets (Fig. 5A). Aiming to extract the universal features of the local binding pathways, we identified residues in each protein that contact the drugs during the binding processes, and their emerging frequencies were mapped onto the structures (Fig. 5B). In addition, the representative pathways obtained from clustering the binding pathways were identified. In all the proteins analyzed, the residues that formed contacts with the drugs were broadly distributed around the target pockets. Based on the residues that frequently formed contacts and the representative binding pathways, 3CLpro, PLpro, and NSP16 seemed to contain the directional preferences in the drug-binding pathways; meanwhile, these preferences were not observed for the other three proteins. In the case of 3CLpro, drugs tended to enter the pocket primarily from the direction of Met49, Gln189, and Asn142. In PLpro, frequent contacts occurred with Leu162, Tyr268, and Gln269. In NSP16, although the largely solvent-exposed DNA-binding groove is likely very accessible, drugs predominantly enter from the opposite side, which contains Leu6898, Asn6899, Asp6912, Cys6913, Ala6914, Gly6946, and Phe6947. The directional preferences of multiple drugs cannot be determined using conventional MD data by observing individual drug–protein interaction, highlighting the significance of analyzing big MD data. These residues might be important for controlling drug access to the target pockets.

**Fig. 6.**
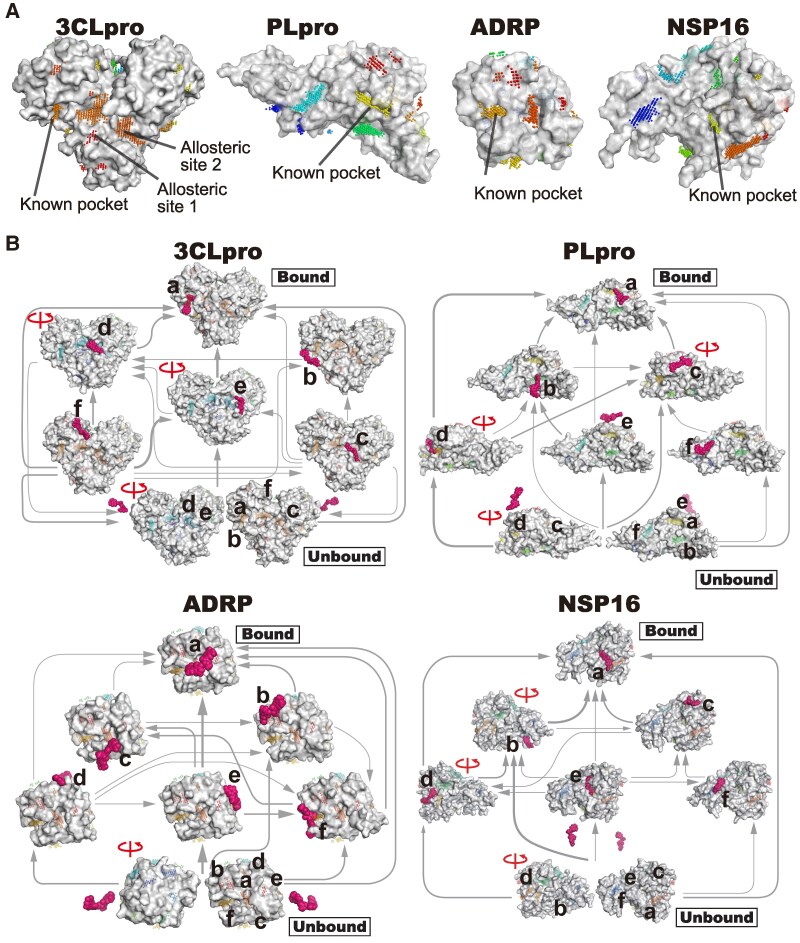
Binding events that occur on the overall protein surfaces. (A) Visualization of the drug-binding sites on the molecular surface for 3CLpro, PLpro, ADRP, and NSP16. The locations where the drugs remained stable are represented by spheres on the 3D structures. Each cluster obtained through the DBSCAN method is represented in different colors. The drug-binding sites found in the previous studies are indicated by black lines and labeled. B) Transition diagrams for eltrombopag olamine derived from the MSM analysis. The MSMs for 3CLpro, PLpro, ADRP, and NSP16 were constructed with seven macro states. Minor flux states are excluded from the diagrams to enhance readability. The width of the arrows interconnecting the states indicates the magnitude of the flux. The rotation axis indicates a view rotated by 180° from the side where the target pocket is visible. The binding sites detected on the molecular surface are indicated by spheres with the same color coding as in (A). The binding sites close to the compound found in each macro state are labeled with lowercase letters, and the label “a” indicates the target pockets. The bound drugs are represented by sphere models colored in magenta.

While the above analyses focused on specific pockets, binding pathways across the entire protein can be analyzed through the MD-based screening approach with ColDock, in which the binding events that occur on the overall molecular surface can be observed. We first extracted the locations at which the evaluated drugs were stably bound on the overall molecular surface. As a result, they were found in extensive regions, including the known active center that was used as target pockets in the above analysis (Figs. 6A and S7). Notably, without using a priori knowledge, we successfully detected allosteric sites in 3CLpro that were determined by previous experiments (25) and the compound-binding site for NSP9 (26), both of which were unknown when COVID-19 first emerged.

We further analyzed the binding processes on the overall protein surfaces of the 10 drugs with the highest multitarget scores using a Markov state model (MSM) (7), which is used to model long-timescale dynamics (Figs. 6B and S8). Here, we focused on eltrombopag olamine, which exhibited broad antiviral activities in various anti-SARS-CoV-2 assays (17) and high multitarget capability toward 3CLpro, PLpro, ADRP, and NSP16 in our simulations (Fig. 3). As shown in Fig. 6B, in addition to transitions from the bulk solvent (unbound state) to the known active pocket (bound state), transitions were observed from the peripheral binding sites identified by the above comprehensive analyses. Additionally, transitions between the peripheral sites were observed. These findings suggest that an extensive transition network is formed among the solvent and the identified binding sites on the protein surface during the compound–protein binding process. These analyses demonstrated that the MD data generated in the present study comprehensively illustrated entire binding processes that occurred over broad timescales, advancing knowledge on the molecular mechanisms that underly compound–protein interactions.

### MD-based screening of 2,105 drugs for 3CL protease to identify promising candidates and perform drug repurposing

**Fig. 7.**
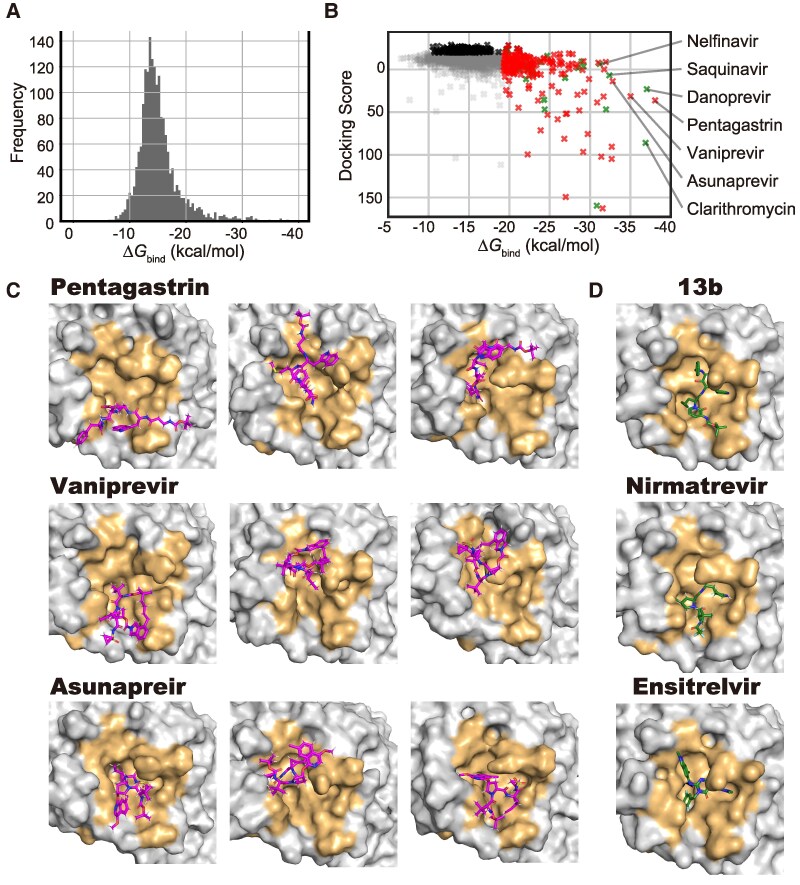
MD-based compound screening of 2,105 commercially available drugs targeting 3CLpro. A) Distribution of Δ*G*_bind_ for the bound 1,844 drugs. B) Correlation between the Δ*G*_bind_ values derived from our MD simulations and the docking scores calculated by the conventional rigid-body docking program rDock (16). Each data point represents an evaluated drug and is indicated by an “×” symbol. The top 200 drugs based on the Δ*G*_bind_ values, the top 200 drugs based on docking scores, and the other points are colored red, black, and gray, respectively. Among the top 200 drugs derived from the MD simulations, drugs that have been investigated in previous preclinical or clinical studies are highlighted with green color. Drugs discussed in the main text are labeled. C and D) In-solution 3CLpro structures complexed with the candidates identified by MD simulations (C) and a structure with known ligands (PDB entry; 6Y2G, 7RFW, and 8DZ0) (D). In the former, the top three-ranked drugs for which preclinical or clinical studies have not been performed were selected as the representative drugs. Multiple binding poses found in the MD simulations are clustered, and the representative poses in the top three clusters with the highest number of members are shown. The MD-derived drugs and known ligands are indicated by the magenta and green stick models, respectively, and amino acid residues within 5 Å of the known ligand in 6Y2G are colored orange.

The achievement of the large-scale spatiotemporal analysis described above prompted us to apply the proposed approach to the drug screening for 3CLpro, a major therapeutic target associated with viral replication. As such, 2,105 commercially available drugs derived from datasets collected by Clarivate were used for drug repurposing, which is an approach used to accelerate a drug development by finding a new therapeutic use for previously approved drugs. Among 2,105 drugs, 1,844 drugs possessed stable binding poses, and a complete list representing the MD-derived binding capabilities was obtained through comprehensively calculating the Δ*G*_bind_ values of the drugs (Table S2). The derived Δ*G*_bind_ values exhibited long-tailed distribution (Fig. 7A), not a narrow distribution, indicating that the drugs with higher affinities toward 3CLpro can be identified by selecting those at the lower end. These results demonstrated that compound screening using the MD-based scores, which has been unrealistic so far, can be achieved with the present workflow.

According to a series of Δ*G*_bind_ values, we subsequently explored the drugs with the potential to bind to 3CLpro with high affinities. Table 1 shows the 20 drugs with the lowest Δ*G*_bind_ values (MD videos can be found at https://clinfo.med.kyoto-u.ac.jp/md_based_screening/si/si2.html). The top candidates included several drugs that were previously considered as promising for COVID-19 treatment based on experimental assays, such as danoprevir (27, 28, 42) (EC_50_ = 87 μM (28)), saquinavir (34, 43, 44) (IC_50_ = 31.4 μM (34)), nelfinavir (36, 37, 45) (IC_50_ = 3.3 μM (37)), lopinavir (46) (EC_50_ = 26.63 μM), asunaprevir (30, 31) (IC_50_ = 15 μM (30)), and vaniprevir (30) (IC_50_ = 6.2 μM). In addition to these antiviral drugs, pentagastrin, a synthetic polypeptide with gastrin-like effects, exhibited the lowest Δ*G*_bind_ values. As the biological function of 3CLpro is to process viral polypeptide as a protease, the identification of the peptide-like drug as a promising candidate is reasonable. Also, another top-ranked candidate, clarithromycin, has been suggested to exhibit its potential antiviral properties (47). In addition to these compounds, several drugs that have not been investigated in preclinical or clinical studies for COVID-19 were included in the top-ranked candidates (Table 1 and Fig. 7B). Given that these drugs are commercially available, they could be new promising candidates to repurpose 3CLpro drugs.

**Table 1.** Top 20 drugs identified in MD-based compound screening for 3CLpro.

Generic name	Δ*G*_bind_ (kcal/mol)	Anti-SARS-CoV-2 activities^a^		Ref.
		In vitro studies	Clinical studies	
Pentagastrin	−38.06			
Danoprevir	−37.03	+	+	(27, 28)
Clarithromycin	−36.88		+	(29)
Vaniprevir	−35.01	+		(28, 30)
Asunaprevir	−32.81	+		(28, 31)
Josamycin	−32.69		+	(32)
Midecamycin	−32.68	+		(33)
Saquinavir	−32.39	+		(34)
Octenidine	−31.98		+	(35)
Telotristat ethyl	−31.94			
Relugolix	−31.74			
Cytarabine	−31.68			
Rifapentine	−31.55			
Solamargine	−31.39			
Nelfinavir	−31.20	+	+	(36–38)
Letermovir	−31.02			
Eptifibatide	−30.88		+	(39)
Digoxin	−30.84	+	+	(40, 41)
Cefotiam hexetil	−29.88			
Rokitamycin	−29.72			

^a^“+” indicates drugs found to have any antiviral effect against SARS-CoV-2, and blanks indicate those with unknown effects based on our investigation of previous reports.

### Advantages of MD-based screening over conventional rigid-body docking simulations

We compared the results obtained from the proposed MD-based approach with those from conventional rigid-body docking simulations (16), revealing substantial differences in the top-ranked drugs: only six drugs were common to the top 200 rankings of both methods (Fig. 7B). Notably, eight drugs that were identified as potential candidates using the MD-based approach—saquinavir, danoprevir, nelfinavir, lopinavir, pentagastrin, asunaprevir, vaniprevir, and clarithromycin—were not included in the top 200 candidates derived from the docking scores. As such, results that cannot be obtained with conventional docking simulations can be obtained with a method that considers dynamic behaviors in a solution. The results of the pairwise RMSD-based clustering of the MD-derived bound structures of the top-ranked drugs revealed that the representative complex structures exhibit multiple structural states with drugs in different binding poses and with target pockets in different shapes (Fig. 7C). In these structures, all or part of the binding region within the known peptide-like inhibitor and drugs was occupied (Fig. 7D), suggesting that these drugs inhibit protease activity by competitively blocking peptide recognition by 3CLpro. This in-solution structural information, which directly corresponds to the observations in real-world situation, cannot be obtained with conventional rigid-body docking simulations.

## Discussion

Despite the substantial interest in large-scale MD-based approaches, these approaches have been impractical because of the required computational resources. In this study, we performed large-scale MD-based spatiotemporal analyses of over 4,000 compound–protein combinations using the state-of-the-art supercomputer Fugaku, demonstrating its potential for investigating molecular recognition mechanisms across diverse types of molecular interactions. Similar to previous MD studies in which millisecond-timescale simulations were performed using a special-purpose machine, Anton (5, 8), our large-scale parallel simulations revealed unique features associated with MD, illustrating whole binding events, including universal features that govern stable interactions and binding processes over broad timescales. Furthermore, we demonstrated the applicability of our proposed method for MD-based drug screening.

The MD simulations performed in this study lasted for 537.9 ms. To complete the equivalent calculations with a conventional GPU-equipped computer, such as the one used in Amazon Web Services (AWS), over 27,000 days are estimated to be required, highlighting the unprecedented scale of this study. Although we successfully completed these calculations over ∼20 days using some of Fugaku's computing resources (21,050 to 21,750 nodes, ∼10 million node-hours in total) that were offered for this study, further reductions in computing costs are needed to enable the routine use of the large-scale spatiotemporal analysis; performing these calculations on AWS (p3.2xlarge on-demand instances) would cost ∼4 million USD. In recent years, the computational power of hardware and applications, including AI and quantum computing technologies, has rapidly advanced. Indeed, by utilizing the new version of the MD program capable of internode parallelism on Fugaku, we are now able to perform ColDock at approximately one-third of the computational cost compared with the time of this study. With these advances, the current MD-based approach is expected to be widely used in the field of structural biology and drug discovery.

The large-scale MD data of drug–protein bindings acquired in this study are unparalleled globally and hold intrinsic value. The data are publicly available at the Biological Structure Model Archive (48) under BSM00058 (https://bsma.pdbj.org/entry/58) and are expected to be valuable for academic purposes and for developing molecular design strategies in future drug discovery studies. Our comprehensive analyses of the big MD data generated provided several insights that are valuable for drug development. For instance, the comprehensive PLIF analyses revealed key residues, such as Arg189 in 3CLpro and Arg166 in PLpro, that are not involved in the recognition of known ligands (Fig. 4). By modifying the ligand structures to interact with these newly found residues, the binding affinity could be improved. Meanwhile, almost all residues identified as essential for the binding processes differed from the key residues that contributed to binding stability, which was determined by analyzing the local binding pathways (Fig. 5B). This observation suggests that a new strategy for drug designs could be based on pathway selectivity, as conventional drug discovery efforts have focused on binding stability. Furthermore, the present approach can identify the multiple drug-binding sites, including experimentally evidenced sites (Figs. 6A and S7). This observation emphasizes the advantage of our approach in identifying target pockets and exploring new ligands based on protein structures when the active pocket is unknown. Notably, only the chemical structures of compounds and the 3D structure of the target protein are needed to gain insights through the MD-based approach, including insights into interaction modes, binding pathways, and binding sites.

Our study revealed that the current approach involves the following limitations: highly lipophilic compounds aggregate, and the bound states of compounds are not captured in some cases. The aggregation of compounds is attributed to the high-concentration environment in ColDock. As suggested in the original paper, this issue can be mitigated by introducing multiple repulsion points into the compounds (14); however, we avoided using this method in the current study to fairly investigate the molecular behavior under the same condition. The limitation in capturing the bound state results from insufficient sampling time in the MD simulations. Improving sampling efficiency is a fundamental issue in MD simulations, and numerous efforts have been dedicated to accelerating the calculations. Further advancements in hardware and applications could address this issue (6, 12, 49). In addition to the challenges associated with the sampling efficiency, whether the commonly used scaling factors in the current LIE approach are appropriate for SARS-CoV-2 proteins is still unclear. We performed a similar grid search using six experimentally validated promising candidates for 3CLpro (danoprevir, saquinavir, nelfinavir, lopinavir, asunaprevir, and vaniprevir) to validate the effectiveness in the screening studies, demonstrating the strongest correlation when using scaling factors identical to those commonly used in this study (Fig. S9A). The calculated Δ*G*_bind_ well-correlated with five compounds, excluding danoprevir (Fig. S9B). These results suggested that the current approach is effective in screenings to explore candidates for target proteins with limited experimental data. The other limitations of the LIE approach include reduced accuracy for systems undergoing large conformational changes upon ligand binding or specific interactions, such as π–π interactions. Additionally, the scaling factors used in this study may be unsuitable for systems dominated by electrostatic interactions. Advancements in force fields and algorithms for more accurate simulations, such as quantum mechanical calculations, could help address these challenges. Combining their developments with ultra-parallel computing on supercomputers could enable more robust and precise simulation-based screening. We think that this study represents a pioneering effort toward such innovative approaches.

We performed a large-scale spatiotemporal analysis that was previously impractical, and further demonstrated that the generated big MD data provided multilateral insights into protein–compound interactions. These results can aid in drug discovery and development. Currently, drug discovery is more challenging as we are facing a shortage of simple drug targets or concepts. Therefore, a large-scale MD-based approach and its resulting insights may provide a new strategy for the development of molecular-targeted drugs.

## Materials and methods

### Selection of compounds for benchmark proteins and MD-based screening

We selected four compounds for each of the benchmark proteins (BACE1, CDK2, CHK1, and HSP90), for which complex structures were previously determined (PDB entry: 3BUH, 3KMX, 3RTH, and 3RTM for BACE1; 4EK4, 4EK5, 4FKL, and 4FKW for CDK2; 4FSY, 4FT7, 4FT9, and 4FTA for CHK1; and 1YC1, 3B27, 2VCI, and 2VHD for HSP90). Additionally, to confirm the generalizability of our approach, we derived an additional 17 compounds for BACE1 (CHEMBL1821798, CHEMBL1821799, CHEMBL1821800, CHEMBL189197, CHEMBL217068, CHEMBL2181988, CHEMBL222533, CHEMBL235825, CHEMBL2437452, CHEMBL2437454, CHEMBL4127062, CHEMBL4127672, CHEMBL4127986, CHEMBL4128807, CHEMBL4128947, CHEMBL568966, and CHEMBL61236) and seven compounds for CDK2 (CID11728093, CID126962160, CID447956, CID5288712, CID5330990, CID6539105, and CID6539304) from the public databases ChEMBL (50) and PubChem (51). Consequently, 40 compound–protein pairs that exhibited variable binding capabilities, in which *K*_d_/*K*_i_ values are ranged from 10^−3^ to 10^−10^ M, were prepared as the initial benchmarking. For the MD-based compound screening targeting 3CLpro, we selected 2,105 commercially available drugs from the list collected by Clarivate. For the MD-based target screening, 355 compounds previously investigated in preclinical or clinical studies were selected from the public portal and data repository created by the Global Health Drug Discovery Institute (GHDDI), which was jointly founded by Tsinghua University, the Bill & Melinda Gates Foundation, and the Beijing Municipal Government (https://ghddi-ailab.github.io/Targeting2019-nCoV). All the drugs evaluated in the MD-based screening are included in Tables S1 and S2.

### Preparation of input files for ColDock calculations

The compounds derived from the databases were preprocessed to add hydrogen atoms at pH 7.0 and remove salts utilizing the Wash function in Molecular Operating Environment (MOE) software, version 2020.09 (Chemical Computing Group, Montreal, Canada). After preprocessing, the 3D conformations of the compounds were generated with the EmbedMultipleConfs tool in Rdkit (https://www.rdkit.org/), and the resulting conformations were optimized with the MMFFOptimizeMoleculeConfs tool in Rdkit. The electrostatic potential of the compounds was calculated at the RHF/6-31G(d)* level using the General Atomic and Molecular Electronic Structure System (GAMESS) program (52), after which the atomic partial charges were assigned by the restrained electrostatic potential approach (53). The initial coordinates of the proteins used in this study were obtained from the PDB (54) (https://www.rcsb.org) and processed using the Structure Preparation module of MOE. Briefly, loops were modeled for disordered regions that contained fewer than seven residues, and other nonnatural N-termini and C-termini were capped with acetyl and formyl groups, respectively. The hydrogen atoms were added, and the topology files were generated using the pdb2gmx module in the GROMACS package (55).

In the benchmark experiments with the compounds for which the complex structures were experimentally determined, the initial models of the proteins were derived from the corresponding PDB entries. For the additional 24 compounds for BACE1 and CDK2, the coordinates in 3RTM and 4FKW were used for the initial protein models, respectively. The initial models for 3CLpro, PLpro, ADRP, NSP16, NSP9, and NSP13 were constructed based on the protein structures in the PDB entries of 6LU7, 6WX4, 6W02, 6W4H, 6WC1, and 6ZSL, respectively. For NSP16, the NSP16–NSP10 dimer was employed as the initial model considering the biological assembly.

### MD simulations

The MD simulations were carried out with periodic boundary conditions (PBCs) using GROMACS 2019.1 on Fugaku (A64FX 48 Core, 158,976 nodes). The Amber ff99SB-ILDN force field (56) was used for proteins and ions, and the general Amber force field (GAFF) (57) was used for compounds. The TIP3P model was used for water (58). The shape of the periodic cell was cubic. Given a compound–protein combination, multiple ligands corresponding to a concentration of 200 mM were randomly placed using the insert molecules module in the GROMACS package. Afterwards, water molecules were placed around the protein model, and 150 mM NaCl was added to neutralize the system. To prevent aggregation, a repulsive force with a Lennard‒Jones potential (*ε* = 10^–3^ kJ/mol, *σ* = 20 Å) was imposed between the pseudoatoms set at the center of mass of the compounds (14). Electrostatic interactions were calculated using the particle mesh Ewald method (59) with a cutoff radius of 10 Å on the direct space, and a nonbonded cutoff of 10 Å was applied for van der Waals interactions. The P-LINCS algorithm was used to constrain all bond lengths to their equilibrium values (60). After minimizing the energy of the fully solvated models, the resulting systems were equilibrated for 100 ps under a constant number of molecules, volume, and temperature conditions, followed by a 100-ps run under a constant number of molecules, pressure, and temperature (NPT) conditions. The temperature was maintained at 298 K by the velocity rescaling with a stochastic term, while the Berendsen pressure coupling was used to maintain the pressure at one bar (61). Subsequently, 100 ns simulations each for 10 different initial distributions of compounds were carried out under NPT conditions without positional restraints. After PBC corrections for the proteins and each compound in the system, the generated trajectories were aligned to the initial model using overall Cα atoms for further analysis. Fugaku is available to researchers worldwide through application and review processes. The code to execute ColDock is found at BSM-Arc under BSM00058 (https://bsma.pdbj.org/entry/58).

### Evaluation of compound aggregation

Aggregations of compounds with an estimated log*P* value of 5 or higher were independently evaluated using MD simulations. Fifty of the compounds were randomly placed inside a 7.5-nm^3^ box filled with TIP3P water without protein. After adding 150 mM NaCl to neutralize the system, we performed 50 ns simulations for the five different initial distributions under the NPT condition (same conditions as the protein–compound simulations). We used the following two metrics calculated from the generated trajectories to evaluate the aggregation: the peak value of the radial distribution function (RDF_max_) calculated by the rdf module in the GROMACS package and a novel metric, *R* (*R* = *N*_ini_/*N*_fin_, where *N*_ini_ and *N*_fin_ are the number of ligand pairs with atomic minimum distances of <1 nm in the initial and final frames of the simulation, respectively). In this study, compounds with *R* < 0.6 or RDF_max_ > 10 were considered aggregated and excluded from subsequent analyses.

### Identification of stable bound states of compounds

Here, we defined the bound state as the compound remaining at the same position for 30 ns or longer. To identify the bound states of compounds, we first determined the positions of the center of mass of the compounds in the voxelized 3D space for all frames obtained in the MD simulations. Subsequently, for a specific frame of interest, if the center of mass remained in nearly the same position for 30 ns or longer, we extracted that frame as a bound state (Fig. S1). The grid spacing was set at 1 Å/grid, and movements within 3 Å of the position in the frame of interest were ignored. The complex structures identified as the bound states were used to calculate the binding free energy and perform structural analyses. Meanwhile, the compounds without bound states were removed from the subsequent structural analyses.

### Extraction of complex structures in the stable bound states at the target pocket

The complex structures in the bound states at the target pocket were identified based on the coordinates of the known ligands in the crystal structures (PDB entry for target protein; 3RTM for BACE1, 4FKW for CDK2, 4FT7 for CHK1, 2VCI for HSP90, 6Y2G for 3CLpro, 6WX4 for PLpro, 6W02 for ADRP, 6W4H for NSP16, and 6XEZ for NSP13). The structures, of which the bound compound's center of mass was assigned to the grid within 3 Å of the ligand coordinates, were extracted and used for the structural analyses as the stable bound states. Regarding NSP9, alpha spheres with the highest propensity for ligand binding score calculated by the Site Finder module in MOE were used to extract the bound states of the complex structures at the target pocket since complex structures with small ligands were not available at the beginning of this study. These analyses were carried out using a molecular-specific programmable environment, high-throughput MD (HTMD) (62), version 1.14.0.

### Calculation of the binding free energy based on the LIE approach

The final binding free energy $\left(\Delta G_{\mathrm{bind}}\right)$ for each drug is an average of the $\Delta G_{\mathrm{bind}}$ values for the complex structures of the bound states. In this study, we applied the LIE approach (15) to estimate $\Delta G_{\mathrm{bind}}$. The LIE approach is an endpoint method that utilizes the bound state of the compound–protein complex at the target pocket and the unbound state under solution conditions. The binding free energy $\Delta G_{\mathrm{bind}}$ is estimated with the following equation:


$$\Delta G_{\mathrm{bind}}=\alpha \Delta V^{\mathrm{vdw}}+\;\beta \Delta V^{\mathrm{ele}}$$

where *α* and *β* are scaling factors for the LIE approach. Here, we adopted α=1.00 and β=0.05, respectively, after a grid search for the values ranging from 0.05 to 1.00 in increments of 0.05, to reproduce the experimentally determined binding affinities referred in the initial benchmark experiments. $\Delta V^{\mathrm{vdw}}$ and $\Delta V^{\mathrm{ele}}$, which are the differences in the average van der Waals and electrostatic interaction energies of the ligand with its environment, are defined as follows:


$$\Delta V^{\mathrm{vdw}}={\left\langle V_{\mathrm{lig}-\mathrm{surr}}^{\mathrm{vdw}}\right\rangle }_{\mathrm{bound}}-{\left\langle V_{\mathrm{lig}-\mathrm{surr}}^{\mathrm{vdw}}\right\rangle }_{\mathrm{unbound}}$$

$$\Delta V^{\mathrm{ele}}={\left\langle V_{\mathrm{lig}-\mathrm{surr}}^{\mathrm{ele}}\right\rangle }_{\mathrm{bound}}-{\left\langle V_{\mathrm{lig}-\mathrm{surr}}^{\mathrm{ele}}\right\rangle }_{\mathrm{unbound}}$$

In the above equations, the average interaction energy ${\left\langle V\right\rangle }_{\mathrm{bound}}$ is estimated from the trajectories generated with ColDock, while the corresponding average energy ${\left\langle V\right\rangle }_{\mathrm{unbound}}$ is estimated based on the trajectories (10 ns × 10 runs) derived from the simulations without the target protein under the same conditions as ColDock. The multitarget score used in target screening is defined as the sum of the Δ*G*_bind_ values for the six SARS-CoV-2 proteins.

### Analysis of PLIFs

In this study, the PLIF values were calculated for all complex structures identified as bound states at the target pockets using the PLIF tool in MOE. The PLIF value for each compound–protein pair can be obtained as an energy-based value with units of kcal/mol. The final PLIF of each drug was calculated by averaging the PLIF values obtained from the associated complex structures, and the summed PLIF values were used to identify the key residues (Fig. S5). The evaluated interaction types include hydrogen bond donors with side chains, hydrogen bond acceptors with side chains, hydrogen bond donors with main chains, hydrogen bond acceptors with main chains, ion interactions, and aromatic contacts, which are denoted as D, A, d, a, I, and R, respectively.

### Analysis of the binding pathways of compounds

The binding pathway was defined as the path taken from the initial contact with the protein’s surface (within 5 Å of the center of mass of drugs) to the closest proximity to the target pocket (within 2 Å of the center of mass of drugs). The extracted pathways were clustered using an X-means clustering algorithm based on the similarity of the time-series data evaluated using the dynamic time warping approach. All drugs except for those exhibiting aggregation were used to identify the binding sites on the overall molecular surface. Based on the frequencies of the bound states in each grid, we extracted grid positions in the top 2% of the frequency range and used the density-based spatial clustering of applications with noise (DBSCAN) method (63) to cluster the extracted positions according to their coordinates. In the clustering algorithm, the minimum number of samples and epsilon value were set to five and 2, respectively. The MSM analysis (7) was performed using the distances between the coordinates of the center of mass of the compound and the atoms of the representative key residues identified in the PLIF analysis (Glu166 for 3CLpro; Asp164 for PLpro; Asp22 for ADRP; and Asp6897 for NSP16) as input features. For dimension reduction, we applied time-lagged independent component analysis (TICA) (64) with a lag time of 5 ns, which involved projecting the input feature vectors onto the six, six, six, and four slowest TICA components for the 3CLpro, PLpro, ADRP, and NSP16 systems, respectively; the results represented 95% of the cumulative kinetic variance. The TICA projection data were then clustered into 1,180, 1,300, 641, and 1,145 clusters for 3CLpro, PLpro, ADRP, and NSP16, respectively, using *k*-means clustering; the number of clusters, *N*_c_, was set to $\sqrt{N_{f}}$, where *N*_f_ is the total number of frames in the input trajectories. For the systems analyzed here, the implied timescales converged beyond 15 ns, which was consistently used for further analysis to construct seven macro states. The analyses were performed with HTMD, version 1.2.4.

## Supplementary Material

pgaf094_Supplementary_Data

## Data Availability

The generated MD data are publicly available at BSM-Arc under BSM00058 (https://bsma.pdbj.org/entry/58). The representative movies after PBC corrections are found in https://clinfo.med.kyoto-u.ac.jp/md_based_screening/si/.
