# Supplementary material for: Precision spatiotemporal analysis of large-scale compound–protein interactions through molecular dynamics simulation
Source: PNAS Nexus. 2025 Mar 22;4(3):pgaf094. doi: 10.1093/pnasnexus/pgaf094 (PMC11949864; doi:10.1093/pnasnexus/pgaf094)
Supplement: pgaf094_Supplementary_Data [file pgaf094_supplementary_data.zip › PNASNEXUS-PNASNEXUS-2024-01401R-s01.docx]

**
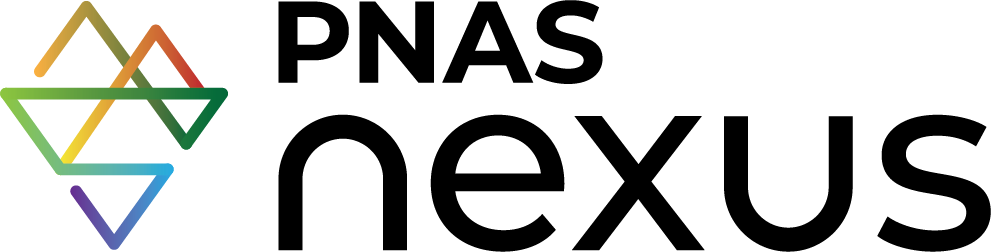
**

**Supplementary Information for**

Precision Spatiotemporal Analysis of Large-scale Compound–Protein Interactions through Molecular Dynamics Simulation

Shigeyuki Matsumoto, Yuta Isaka, Ryo Kanada, Biao Ma, Mitsugu Araki, Shuntaro Chiba, Atsushi Tokuhisa, Hiroaki Iwata, Shoichi Ishida, Yoshinobu Akinaga, Kei Terayama, Ryosuke Kojima, Yohei Harada, Kazuhiro Takemura, Teruki Honma, Akio Kitao, Yasushi Okuno

Yasushi Okuno

Email**:**  okuno.yasushi.4c@kyoto-u.ac.jp

**This PDF file includes:**

Figures S1 to S9

Legends for Movies S1 to S2

Legends for Tables S1 to S2

**Other supporting materials for this manuscript include the following:**

Movies S1 to S2

Tables S1 to S2


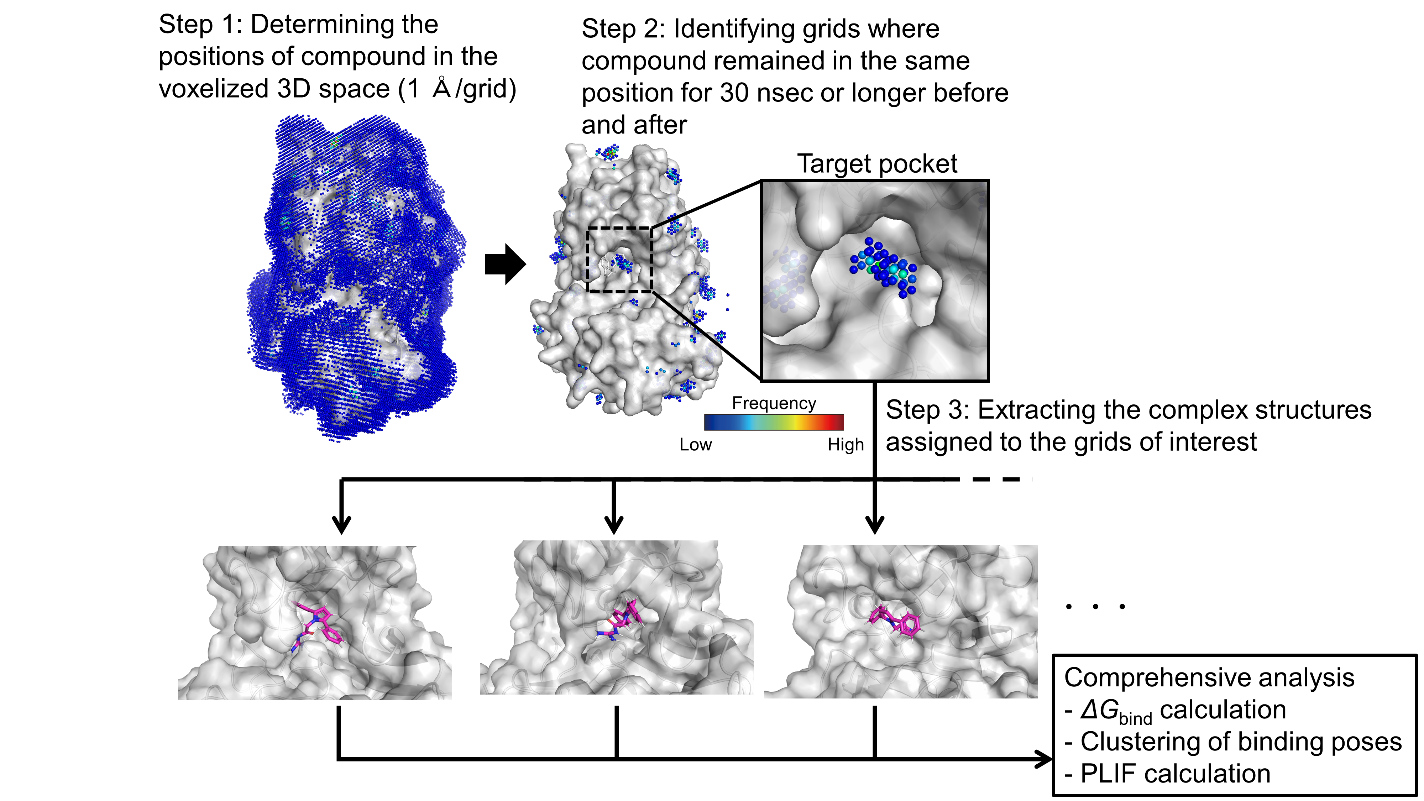


**Fig. S1**. **Workflow for identifying the complex structures in the stably bound state.** The positions for the center of mass of the compounds in the voxelized 3D space were determined from the MD data. Among them, the frames in which the compound’s center of mass remained in the same position for 30 ns or longer before and after were extracted as a bound state. After the complex structures that were assigned to the grids of interest were extracted, the structures were used for subsequent analyses, including the Δ*G*_bind_ calculation, clustering of binding poses, and PLIF calculation. In the image of step 1, the grids that representing the location of the compound’s center of mass in the voxelized 3D space are indicated by sphere models, and they are colored with a rainbow gradient that ranges from blue (low) to red (high) according to the frequencies found. In the image of step 2, sphere models similarly indicate the positions of the center of mass of the compounds assigned as the bound state. In the images of step 3, the bound compounds are represented by stick models. For this visualization, the simulation results in the BACE1 and ChEMBL217068 systems are used as an example.

**
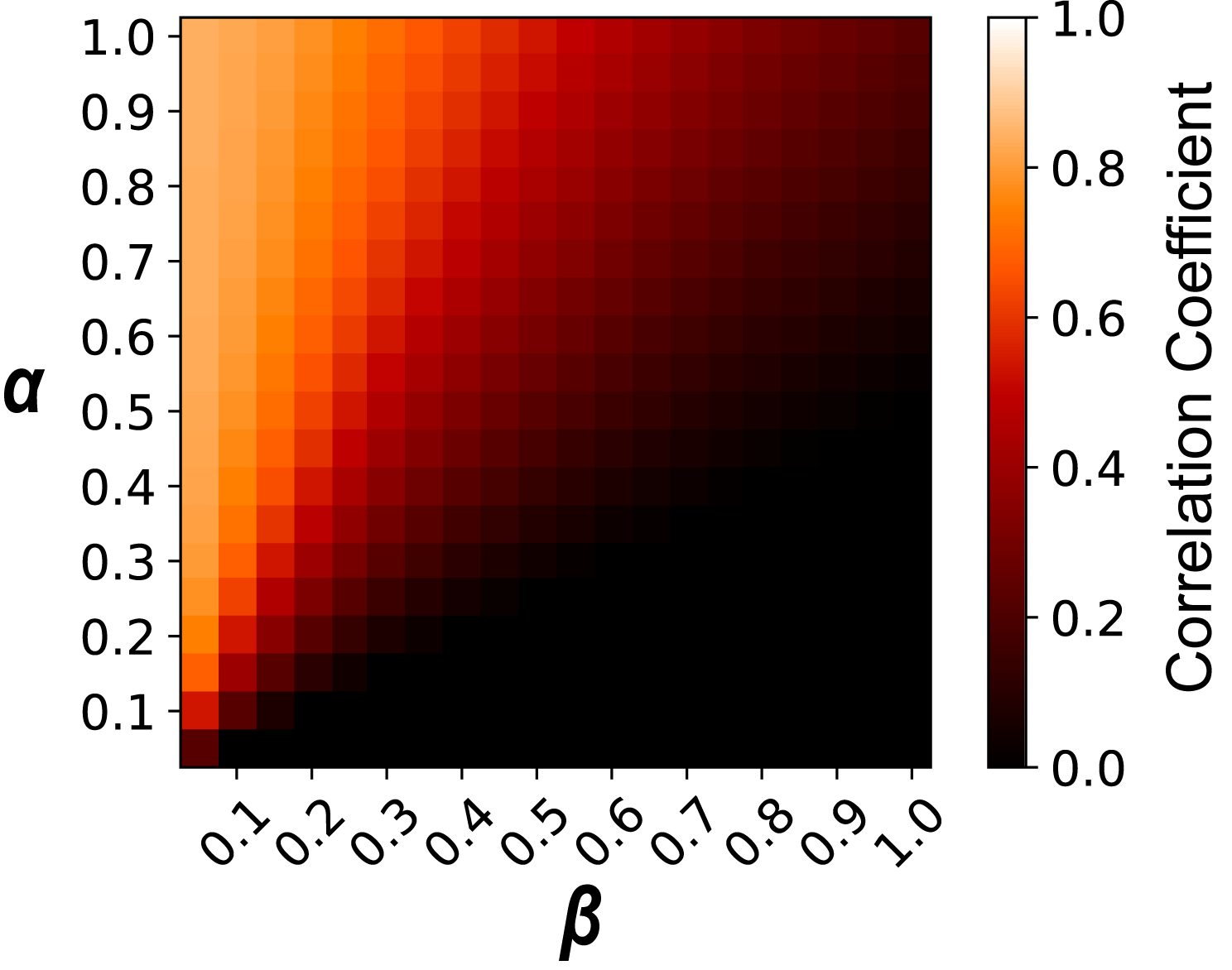
**

**Fig. S2. Exploring the most appropriate scaling factor for Δ*G*_bind_ calculation with LIE approach.** The scaling factor was explored via a grid search for the values ranging from 0.05 to 1.00 in increments of 0.05 across four benchmark proteins (26 protein–compound pairs). $\alpha$ and $\beta$ represent the scaling factors for ${\Delta V}^{\mathrm{vdw}}$ and ${\Delta V}^{\mathrm{ele}}$, respectively. The correlation coefficients between experimentally determined Δ*G*_bind_ values and those calculated with the indicated $\alpha$ and $\beta$ values are visualized as a heatmap.

**
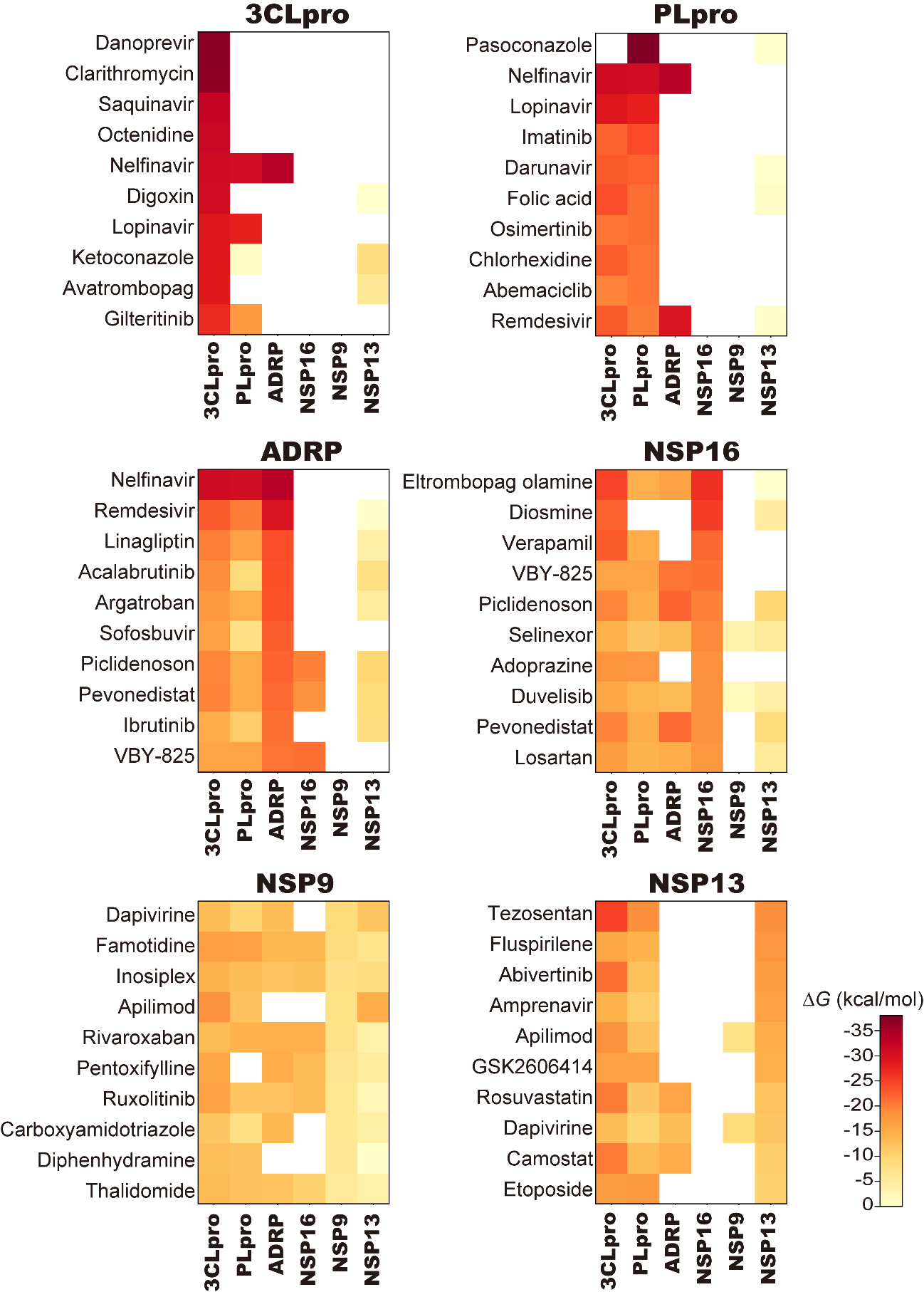
**

**Fig. S3. Top 10 compounds according to the Δ*G*_bind_ for each of the six SARS-CoV-2 proteins.** The Δ*G*_bind_ values of the top 10 compounds are shown on a heatmap, along with those for the other proteins. The horizontal axis represents proteins, and the vertical axis represents compounds. Combinations for which no binding was observed are shown in white.


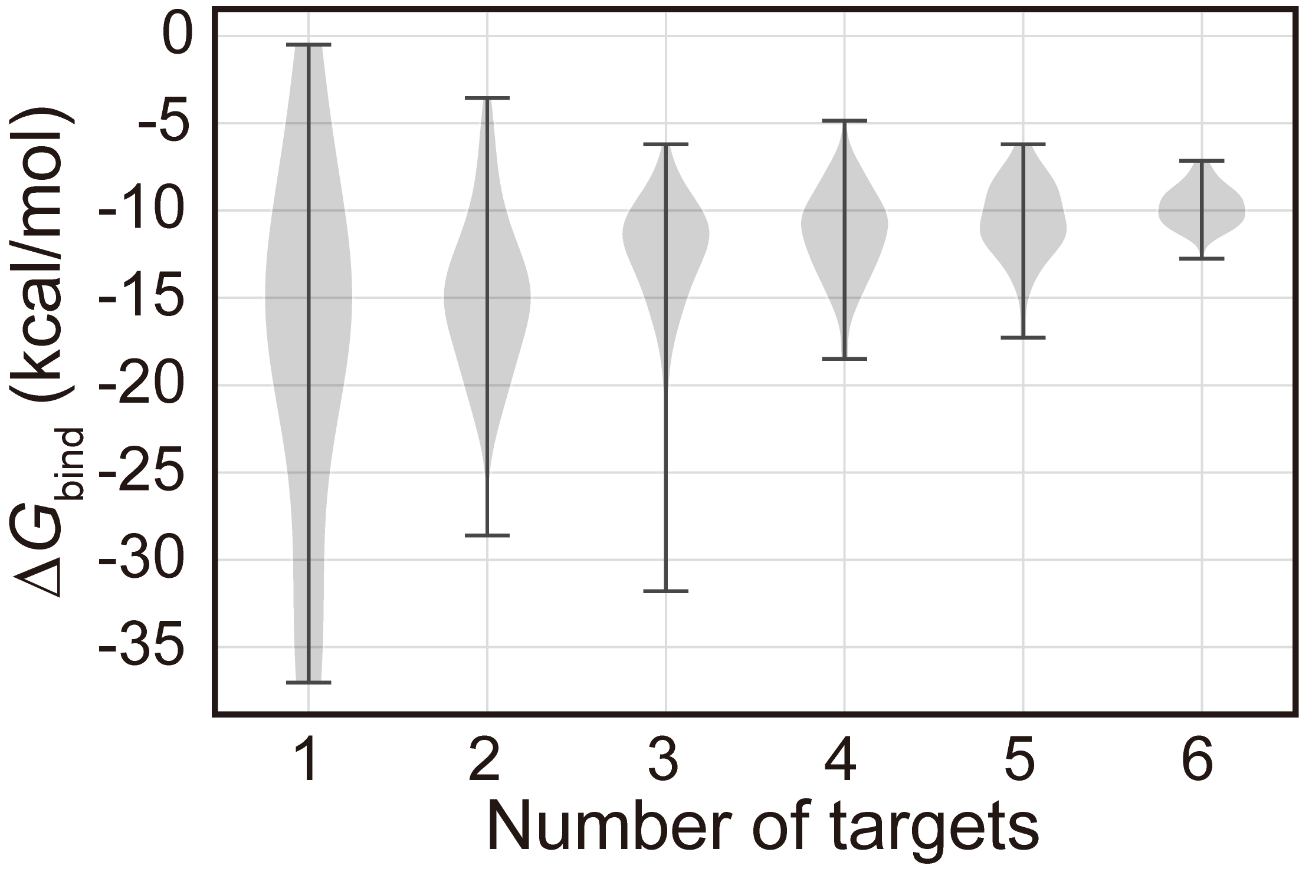


**Fig. S4. Relationship between multitarget capability and Δ*G*_bind_.** The distribution of average Δ*G*_bind_ for each drug against the number of targets is shown in a violin plot. The horizontal axis represents the number of targets, and the vertical axis represents the Δ*G*_bind_ value of each drug averaged over the number of targets.

**
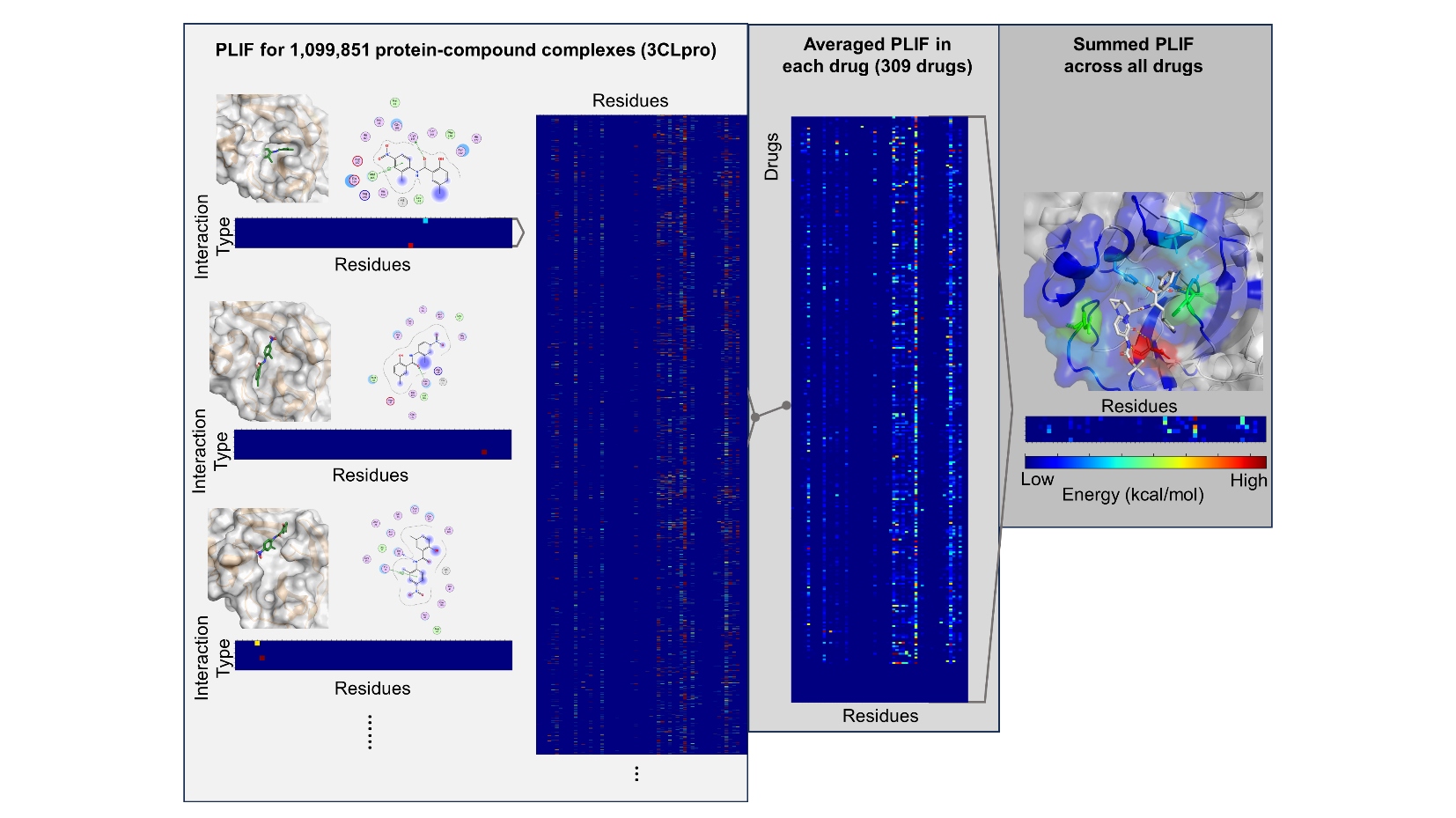
**

**Fig. S5. Workflow for the comprehensive analysis of PLIFs.** PLIFs were calculated for all protein-compound complex structures identified as the bound states (left panel). The energy-based PLIF data are shown as a heatmap with a rainbow gradient ranging from blue (low) to red (high). In the left panel, the 3D structures to be calculated and the 2D protein–compound interaction diagrams are shown together with the heatmaps. The PLIFs for each drug were obtained by averaging the PLIFs derived from the associated complex structures (middle panel). The sum across all drugs was used for identifying key residues and was mapped onto the 3D structure with a similar color gradient (right panel), in which the known ligand and the residues that exhibit higher energies are represented by a stick model. In the heatmap, the horizontal axis represents the residues, and the vertical axis represents the complex structures, drugs, or interaction types. For this visualization, the MD data for 3CLpro were used as an example.


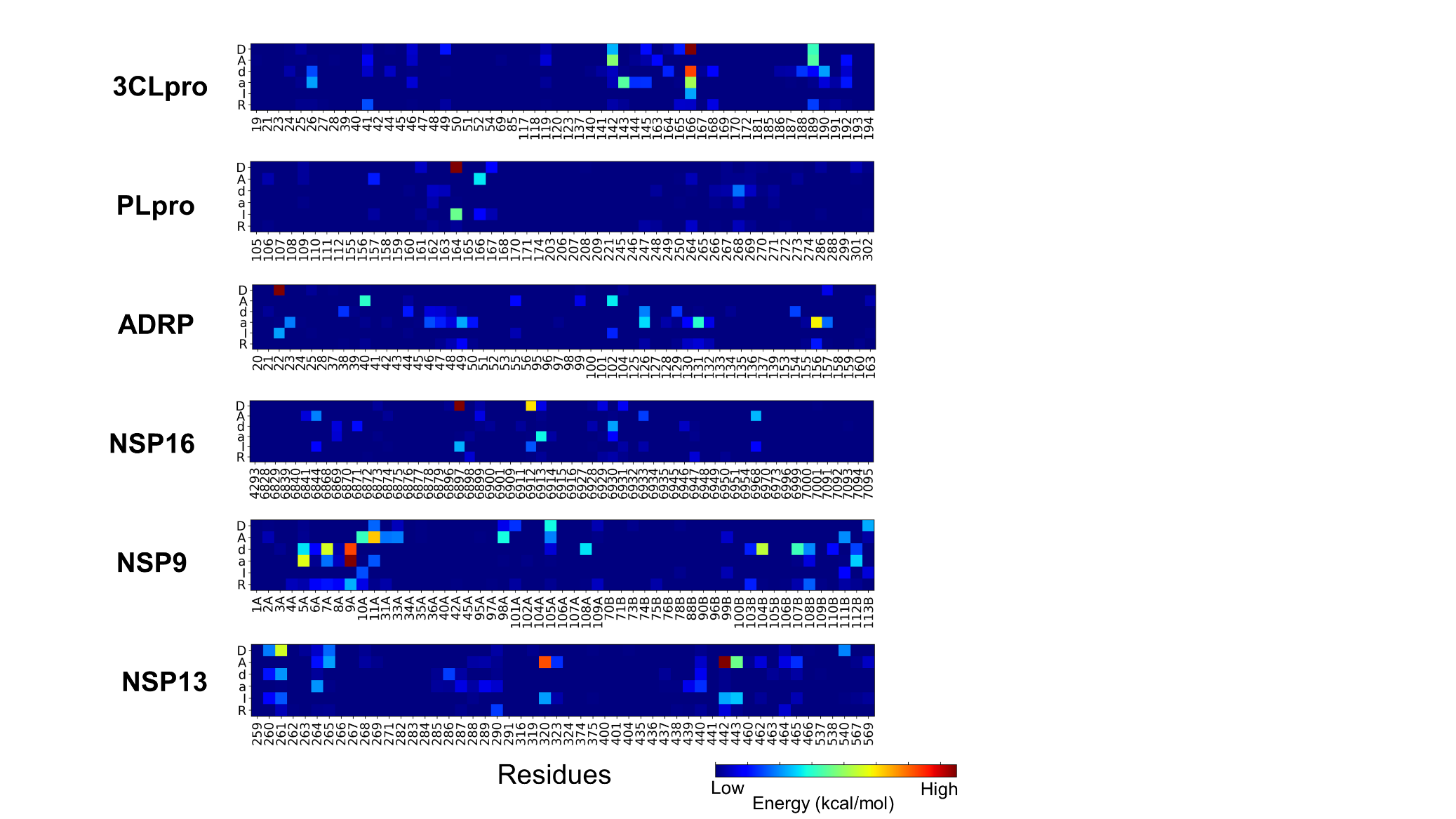


**Fig. S6. PLIF across all drugs in each protein.** The sums of energy-based PLIF are shown as heatmap with a gradient of rainbow ranging from blue (low) to red (high). The horizontal axis represents the residues, and the vertical axis represents the interaction types, in which hydrogen bond donor with side chain, hydrogen bond acceptor with side chain, hydrogen bond donor with main chain, hydrogen bond acceptor with main chain, ion interaction, and aromatic contact are denoted as D, A, d, a, I, and R, respectively. For NSP9, the chain id is labelled with the residue number since the biological assembly involves a dimer.


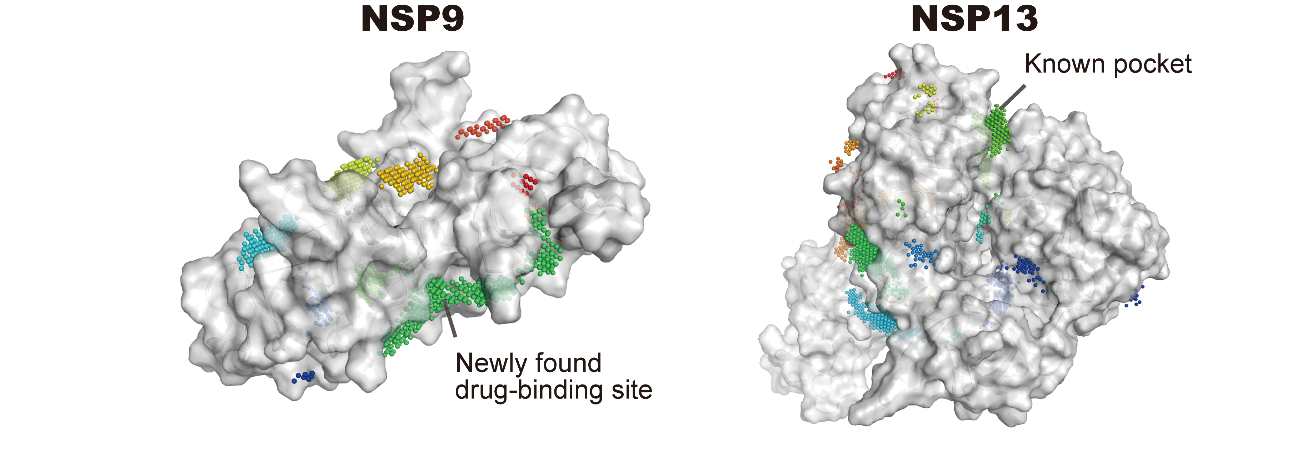


**Fig. S7. Visualization of the drug binding sites on the molecular surface for NSP9 and NSP13.** The locations where the drugs remained stable are represented by spheres on the 3D structures. Each cluster obtained through the density-based spatial clustering of applications with noise (DBSCAN) method is represented in different colors. The drug-binding sites found in the previous studies are indicated by black lines and labelled.


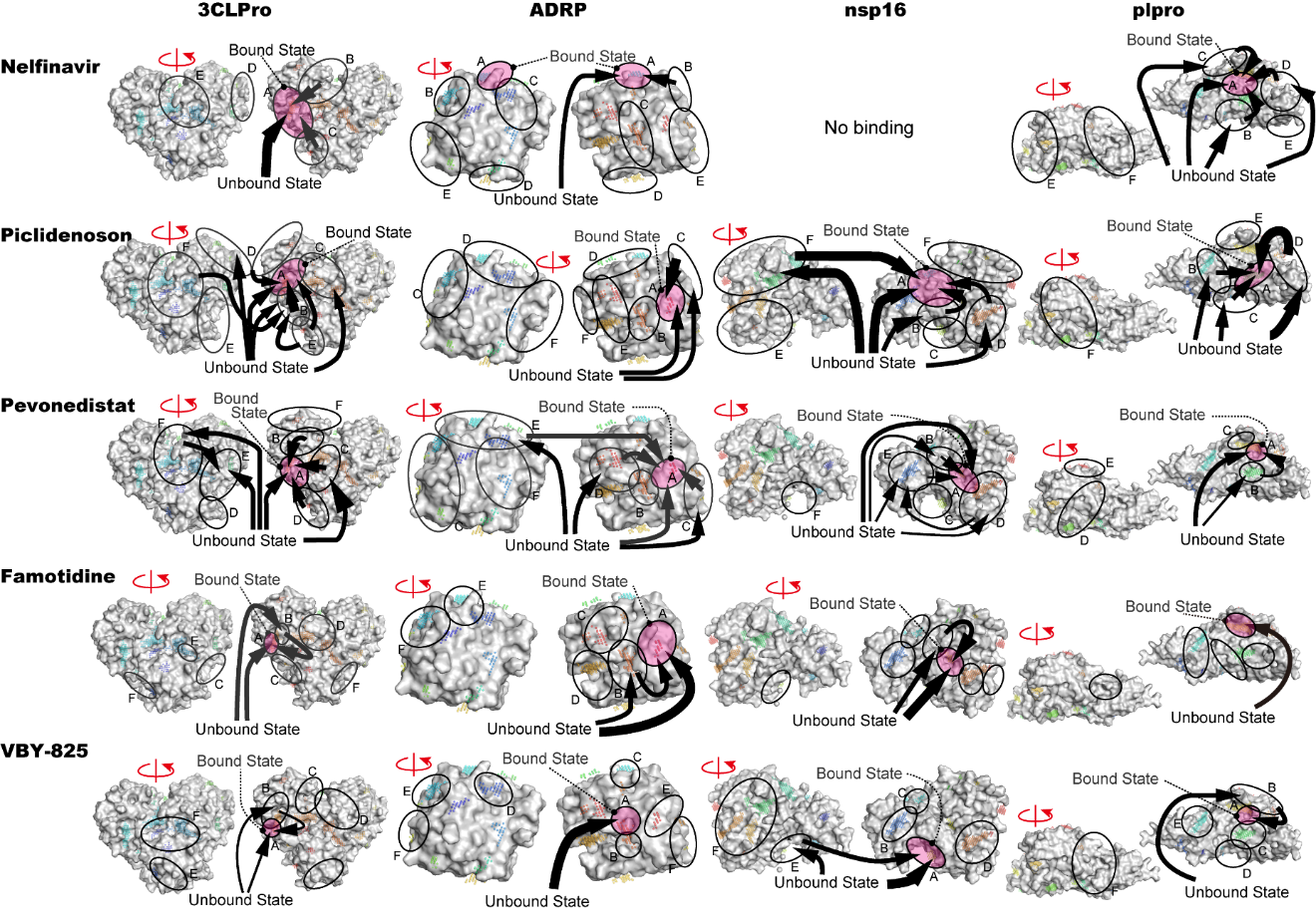


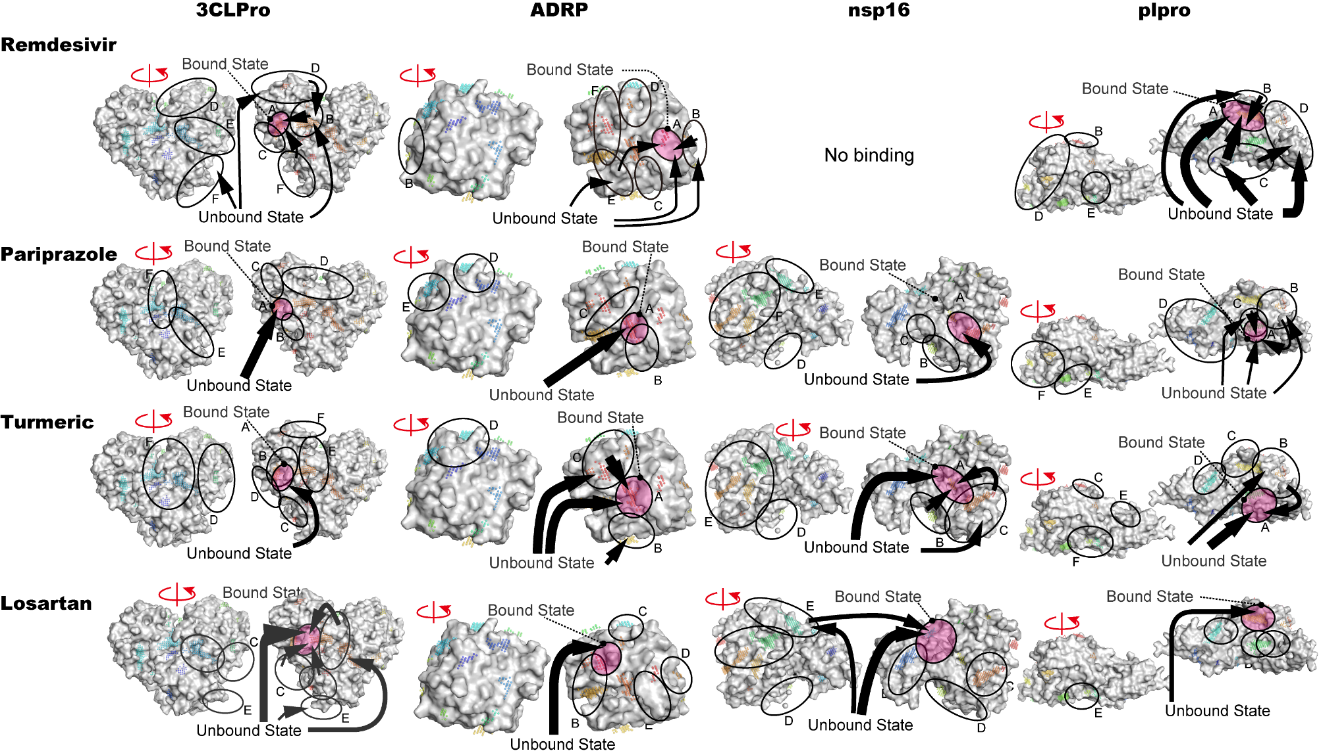


**Fig. S8. Transition diagram derived from MSM analysis for compounds with high multitarget scores.** MSM models for 3CLpro, PLpro, ADRP, and NSP16 are constructed with seven macro states. Minor flux states are excluded from the diagrams to enhance the readabilities. The width of the arrows interconnecting the states indicates the magnitude of the flux. The rotation axis indicates a region rotated 180 degrees from the side at which the target pocket is visible. The binding sites detected on the molecular surface are indicated by spheres, the colors of which are identical to those in Fig. 7A of the main text. The binding sites close to the compound found in each macrostate are labelled with uppercase letters, and the label “A” indicates the target pockets.

**
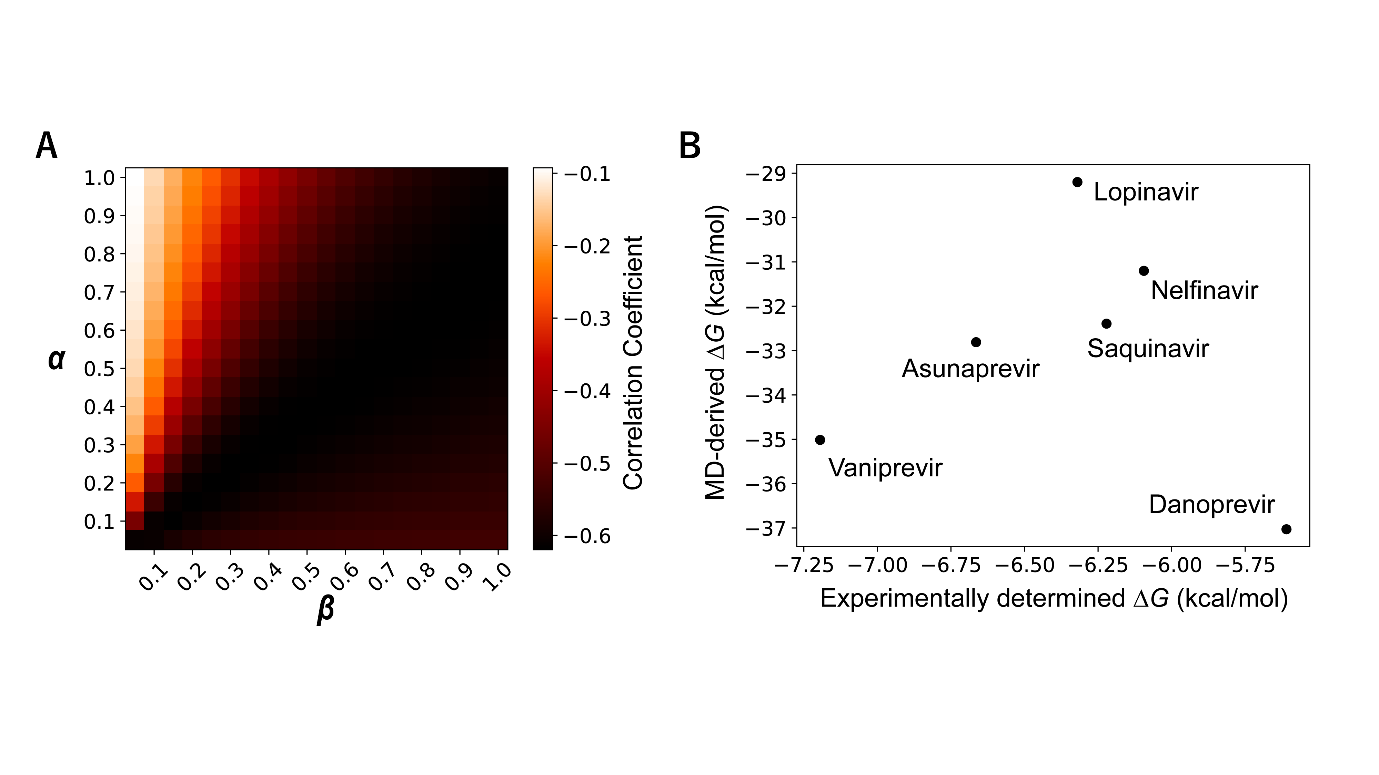
**

**Fig. S9. Evaluation of the validity of the scaling factors used in the screening studies.** (A) Exploring the most appropriate scaling factors for Δ*G*_bind_ calculation with the LIE approach in 3CLpro. The scaling factor was explored via a grid search for the values ranging from 0.05 to 1.00 in increments of 0.05 using six experimentally validated promising candidates. $\alpha$ and $\beta$ represent the scaling factors for ${\Delta V}^{\mathrm{vdw}}$ and ${\Delta V}^{\mathrm{ele}}$, respectively. The correlation coefficients between experimentally determined Δ*G*_bind_ values and those calculated with the indicated $\alpha$ and $\beta$ values are visualized as a heatmap. (B) Correlation between the experimentally determined Δ*G*_bind_ and those derived from the MD simulations. Each point represents a compound–protein pair and is labeled with the drug name.

**Movie S1 (separate file). Overview of a movie of ColDock.** A protein and the concentrated compounds are represented by surface and stick models, respectively. A focused pocket is colored by yellow.

**Movie S2 (separate file). Close-up view of a movie of ColDock.** A protein and the concentrated compounds are represented by surface and stick models, respectively. A focused pocket is colored by yellow.

**Table S1 (separate file). A complete list representing the MD-derived binding capabilities of compounds evaluated in spatiotemporal analysis of multiple interactions.**

**Table S2 (separate file). A complete list representing the MD-derived binding capabilities of compounds evaluated in MD-based drug screening for 3CL protease.**
